# Prevalence of vertebral fractures in women and men in the population-based Tromsø Study

**DOI:** 10.1186/1471-2474-13-3

**Published:** 2012-01-17

**Authors:** Svanhild Waterloo, Luai A Ahmed, Jacqueline R Center, John A Eisman, Bente Morseth, Nguyen D Nguyen, Tuan Nguyen, Anne J Sogaard, Nina Emaus

**Affiliations:** 1 Department of Community Medicine, Faculty of Health Sciences, University of Tromsø, 9037 Tromsø, Norway; 2 Department of Health and Care Sciences, Faculty of Health Sciences, University of Tromsø, 9037 Tromsø, Norway; 3 Garvan Institute of Medical Research, University of New South Wales, Sydney, Australia; 4 National Public Health Institute, Oslo, Norway

**Keywords:** Morphometry, Vertebral deformity, Vertebral fractures, Population-based study

## Abstract

**Background:**

Osteoporotic vertebral fractures are, as the hip fractures, associated with increased morbidity and mortality. Norway has one of the highest reported incidences of hip fractures in the world. Because of methodological challenges, vertebral fractures are not extensively studied. The aim of this population based study was to describe, for the first time, the age- and sex specific occurrence of osteoporotic vertebral fractures in Norway.

**Methods:**

Data was collected in the Tromso Study, 2007/8 survey. By the use of dual x-ray absorptiometry (GE Lunar Prodigy) vertebral fracture assessments were performed in 2887 women and men aged from 38 to 87 years, in addition to measurements of bone mineral density at the femoral sites. Information on lifestyle was collected through questionnaires. Comparisons between fractures and non-fractures were done sex stratified, by univariate analyses, adjusting for age when relevant.

**Results:**

The prevalence of vertebral fractures varied from about 3% in the age group below 60 to about 19% in the 70+ group in women, and from 7.5% to about 20% in men, with an overall prevalence of 11.8% in women and 13.8% in men (*p *= 0.07). Among those with fractures, only one fracture was the most common; two and more fractures were present in approximately 30% of the cases. Fractures were seen from the fourth lumbar to the fifth thoracic vertebrae, most common between first lumbar and sixth thoracic vertebrae. The most common type of fracture was the wedge type in both sexes. Bone mineral density at the hip differed significantly according to type of fracture, being highest in those with wedge fractures and lowest in those with compression fractures.

**Conclusions:**

The prevalence of vertebral fractures increased by age in women and men, but the overall prevalence was lower than expected, considering the high prevalence of hip and forearm fractures in Norway. In both sexes, the wedge type was the fracture type most frequently observed and most common in the thoracic region.

## Background

Osteoporosis and osteoporotic fractures occur so commonly worldwide, they are a serious health issue [1,2]. Forearm, vertebral and hip fractures are reportedly the most frequent osteoporotic fractures [3]. Whereas hip fractures are the most costly because of the expenses for treatment and rehabilitation imposed on society [4], many publications indicate that vertebral fractures are the most common form of osteoporotic fractures [5-7]. However, limited data support this claim. Some studies report that only one in three vertebral fractures are diagnosed [6,8] and as such argue that vertebral fractures are largely under diagnosed [9-11].

Osteoporotic vertebral fractures--meaning fractures in one or more vertebrae--are, as the hip fractures, associated with increased morbidity [12] and mortality [13-15]. Having one vertebral fracture also significantly increases the risk of experiencing subsequent vertebral fractures [16-18], as well as other fractures [9,19-21]. In general, we have less knowledge about vertebral fractures than about other fractures because of the methodological problems related to their verification. Prevalence data from population based studies indicate a substantial variation in overall prevalence ranging from 10% to 25% in women and from 10% to 27% in men [4,5,17,22-30]. Prevalence data from patient studies [31-34] are not suited to define prevalence in a general population, because the health states associated with "patient" status may affect the risk and frequency of fractures. Several studies report that Norway is among the countries in the world with the highest rate of osteoporotic fractures, including hip [35] and forearm [36]. Until now, prevalence data on vertebral fractures have not been presented from any major population based study in Norway. Thus, knowledge and data on the frequency of vertebral fractures in a country with reported high rates of other osteoporotic fractures is warranted.

Data presented here are generated in the population based Tromsø Study (2007/08 survey). The aims of the study are to describe the age related rate of vertebral fractures in men and women and to examine which type of fracture is the most common, which vertebrae are the most prone to fractures, as well as the severity of these fractures.

## Methods

### Study population

The Tromsø Study is a longitudinal population based multi-purposed study focusing on lifestyle related diseases, comprising six repeated surveys and examinations starting in 1974 (Tromsø I) and repeated in 1979/80, 1986/87, 1994/95, 2001/02 and 2007/08 (Tromsø VI) [37]. Only men were invited to the first survey which focused on cardiovascular diseases, but from Tromsø II 1979/80 both women and men have been included. The participation rate has ranged from 65% to 77% [38]. Each survey has been conducted in two phases, with the most basic examination in phase 1 (height, weight, BP, blood samples, and questionnaires) and more extensive examinations for a random sub-sample of the cohort in phase 2, depending on available resources.

**Figure 1:**
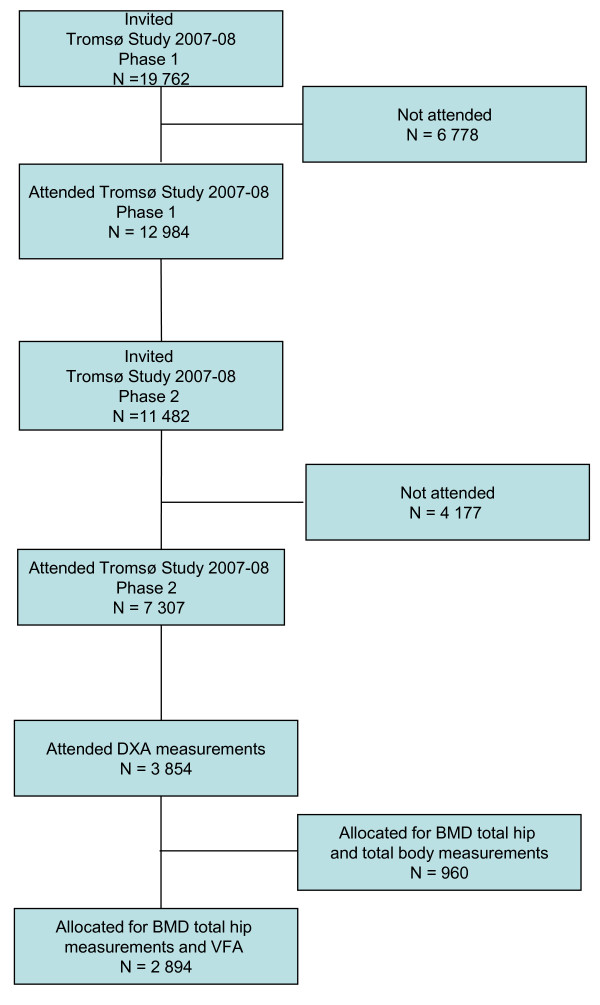
**Study profile**.

For Tromsø VI, in total 19 762 subjects were invited and 12 984 (65.7%) attended phase 1, 6054 men (62.9%) and 6930 women (68.4%). Among those participating in phase 1, a total of 11 484 subjects were invited for phase 2, and 7307 (64%) attended, 3141 men (61.5%) and 4166 women (65.3%). Among those attending phase 2, only persons with valid bone mineral density (BMD) measurement from Tromsø V in 2001/2002 were invited for a Dual X-ray (DXA) BMD measurement of the hip, i.e. a dual femur scan, and altogether 3854 persons attended. Among these, a lateral vertebral assessment (LVA), hereafter called vertebral fracture assessment (VFA) was performed in a randomly selected group of 2894 persons (Figure 1). Seven blurred VFA scans had to be excluded, leaving 2887 persons, 1681 women and 1206 men, with clearly measurable VFA scans and total hip measurements.

### Measurements

Vertebral morphometry is a quantitative method developed for identification of osteoporotic vertebral fractures based on the measurement of vertebral heights. Although spine radiographs are generally considered to be the gold standard for the diagnosis of vertebral fractures [20,39], the morphometric method is recognized for being easy, precise and using low radiation exposure [40-42]. When combined with BMD measurements, it is even argued it could become the "gold standard" [43]. Determination of fracture types was done visually according to a standard set by GE Lunar Prodigy, also shown in Kim et al. [11]. Three types of fractures are identified: wedge, biconcave, and compression, according to three degrees of severity, ranging from mild through moderate to severe [40]. The wedge fractures are characterized by deformed structure of the anterior part of the vertebrae, the biconcave of the middle part, and the compression of the total vertebrae. All our scans were taken according to a standard set by GE Lunar Prodigy, Lunar Corp., Madison, USA, and in GE Lunar encore version 12.20. Daily phantom measurements were performed throughout the survey. Specially trained technicians did the scanning according to the standardized protocol, and one of them performed the quality assessment of the total material afterwards. In a recent validation study, the short term in vivo precision error for the Lunar Prodigy was 1.7% and 1.2% for the femoral neck and total hip measurements, respectively [44]. For precision analysis of the VFA, random sample of 50 participants was reanalyzed. The mean intra-class correlation coefficient was 0.82, 0.79, 0.82, and 0.84 for anterior, middle, posterior, and average height, respectively, all vertebrae considered. At the vertebrae with highest frequency of present deformity, exemplified by 7^th ^and 12^th ^thoracic vertebrae, the intra-class correlation coefficient varied between 0.77 and 0.92, with a mean of 0.86. Additional measurements taken were dual hip BMD expressed as g/cm^2^, and height and weight, in light clothing without shoes, were measured in all the participants.

### Questionnaire

Information on lifestyle variables was collected through questionnaires in both phases of the study. The question on smoking had three alternatives: present, former, and never. These were grouped into two, where former and never smokers were categorized as "not smoking" and smokers as "smoking". The question on physical activity had four alternatives, from sedentary through moderate and high to very high physical activity level each week. Having few answers both in the "sedentary" and "very high physical activity level" groups, we categorized sedentary and moderate physical activity level as moderate, and high/very high level as high. Five levels of self-perceived health (very good, good, neither nor, bad, very bad) were categorized into two, good (very good and good) and poor. Educational information was combined from five to three levels: primary school only (i.e. 7 years), O-level, and more than O-level. Our study population being rather old, primary school only was not uncommon (Table 1).

**Table 1:** Descriptive statistics by gender and morphometric vertebral fracture, the Tromsø Study 2007-08

Gender and factor	No Fracture	Vertebral fracture	*P*-value
Men (N)	1040	166	
Age	64.8 (9.3)	69.0 (9.2)	< 0.0001
Weight, kg	84.5 (12.3)	82.8 (11.5)	0.078
Height, cm	175.5 (6.5)	174.4 (6.7)	0.062
BMI, kg/m^2^	27.4 (3.5)	27.2 (3.4)	0.457
Bone mineral density, total hip (g/cm^2^)	1.03 (0.14) n = 1007	0.97 (0.15) n = 160	< 0.0001
**Education**			0.147
1 (n; %)	324 (31.9)	54 (33.5)	
2	287 (28.2)	55 (34.2)	
3	405 (39.9)	52 (32.3)	
**Physical activity**			0.577
High active (n; %)	213 (22.6)	31 (20.5)	
Low active	731 (77.4)	120 (79.5)	
**Smoking status**			0.988
Daily smokers (n; %)	159 (15.5)	25 (15.5)	
Non smoking	868 (84.5)	136 (84.5)	
**Health status**			0.574
Good (n; %)	662 (64.1)	102 (61.8)	
Poor	371 (35.9)	63 (38.2)	

**Women (N)**	**1482**	**199**	

Age	64.7 (9.3)	70.5 (8.6)	< 0.0001
Weight, kg	71.1 (12.5)	68.4 (12.7)	0.005
Height, cm	162.5 (6.3)	160.4 (7.1)	< 0.0001
BMI, kg/m^2^	26.9 (4.6)	26.6 (4.5)	0.297
Bone mineral density, total hip (g/cm^2^)	0.91 (0.13) N = 1392	0.83 (0.11) n = 179	< 0.0001
**Education**			0.009
1 (primary school) (n; %)	627 (43.0)	106 (54.4)	
2 (O-level)	375 (25.7)	44 (22.6)	
3 (more than O-level)	456 (31.3)	45 (23.1)	
**Physical activity**			0.796
High active (n; %)	144 (11.2)	18 (10.5)	
Low active	1143 (88.8)	153 (89.5)	
**Smoking status**			0.750
Daily smokers (n; %)	265 (18)	37 (19)	
Non smoking	1189 (82)	156 (81)	
**Health status**			0.029
Good (n; %)	905 (61.9)	106 (53.8)	
Poor	557 (38.1)	91 (46.2)	

*P-values refer to univariate analyses

### Statistics

Baseline characteristics in women and men with and without fractures were compared by univariate analyses, using Independent sample T-test for continuous variables and chi-square testing for categorical variables. To adjust for age differences, logistic regression was applied to test the differences of the significant variables between the groups. Prevalence of morphometric fractures in women and men was compared by chi square testing, and so was distribution of deformities and types of deformities (wedge, biconcave, or compression). The mean BMD difference between the three different types of deformities was tested in both sexes using ANOVA, adjusting for age. The statistical analyses were performed by SPSS version 18, and a *p*-value below 0.05 was considered significant.

The Regional Committee of Research Ethics recommended the study, and written informed consent was obtained from all participants.

## Results

The study cohort comprises 2887 women and men ranging from 38 to 87 years of age (Table 1). Vertebral deformities were present in 199 women and 166 men. Women with vertebral fractures were older, shorter, weighted less, had lower total hip BMD, lower educational level, and lower self-reported health compared to those without fractures. When adjusting for age, only weight and BMD were significantly different between the groups. Men with vertebral fractures were older and had lower BMD compared to those without fractures. Adjusting for age, BMD remained significantly different between the groups (Table 1). With an overall prevalence of any deformity of 11.8% in women and 13.8% in men, the prevalence was not significantly different between the sexes (*p *= 0.07) (Table 2). In both sexes, the prevalence increased significantly by age, in women from 3.4% below the age of 60 to 19.2% over 70 years, in men from 7.6% to 20.3%, respectively (Table 2). The prevalence was not significantly different between the sexes in the age groups 60-69 years (*p *= 0.37), 70+ years (*p *= 0.36), but was higher in men in the age group below 60 years (*p *= 0.008).

**Table 2:** Prevalence of morphometric vertebral fracture by age in women and men

Vertebral fracture	Men		Women	
	Fracture/N	Prevalence (%)	Fracture/N	Prevalence (%)
Any fracture	166/1206	13.8	199/1681	11.8
95% CI		(11.9, 15.8)		(10.4, 13.5)

**Age group**				

< 60	26/342	7.6	14/412	3.4
60-69	50/420	11.9	80/721	11.1
70+	90/444	20.3	105/548	19.2

**Figure 2:**
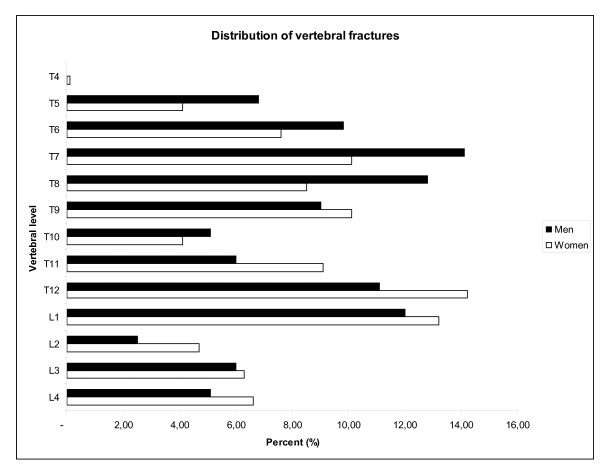
**Percentage distribution of vertebral deformities (all types) according to vertebral level in 1681 women (317 deformities) and 1206 men (234 deformities)**. Calculations based on the following number of measurements: T4 = 2350, T5 = 2743, T6 = 2845, T7 = 2863, T8 = 2875, T9 = 2878, T10 = 2885, T11-L3 = 2887, L4 = 2848.

**Figure 3:**
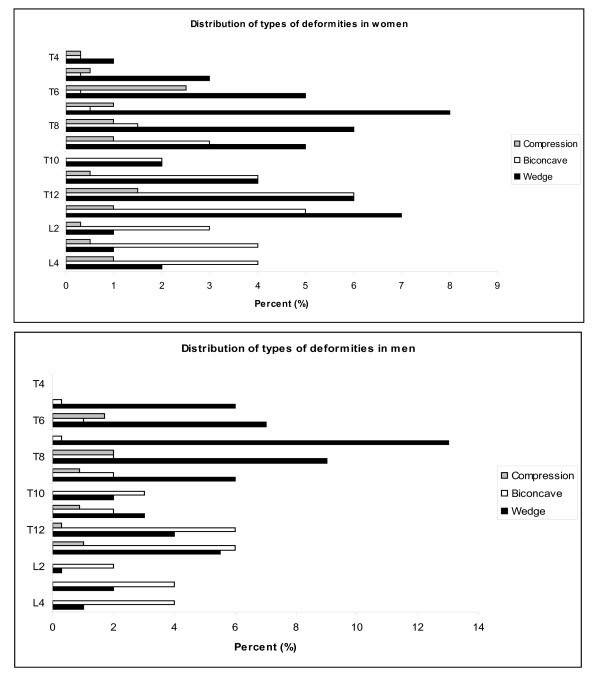
**Distribution of types of vertebral deformities according to vertebral level in A. 1681 women and B. 1206 men**.

Numbers of deformities varied from 1 to 6 in each person so that in total there were 317 and 234 deformities in women and men, respectively. The distribution of numbers of deformities, categorized into 0, 1, 2, 3 or more deformities, was not significantly different between the sexes (*p *= 0.169) (Table 3). In both sexes, more than 95% of the deformities were either moderate or severe wedge, moderate or severe biconcave or moderate compression (Table 4). Types of deformities differed significantly between the sexes (*p *= 0.025). In women, half (51%) of the deformities were wedges (moderate or severe), more than one third (37%) were biconcavities (mild, moderate, or severe), and 12% were compressions (moderate or severe). In men, the proportions with the last two fracture types were somewhat lower (33% and 6%), whereas the proportions with wedge deformities were higher (60%) than in women (Table 4). The distribution of the locations of the deformities showed a similar pattern in men and women (Figure 2). In women, the majority of deformities, defined as more than 10% of the total deformities, were at 7^th^, 9^th^, 12^th ^thoracic and 1^st ^lumbar vertebrae, in men at 7^th^, 8^th^, 12^th ^thoracic, and 1^st ^lumbar vertebrae (Figure 3A and 3B). In both sexes, most of the wedge deformities were present from 12^th ^thoracic and above, and most of the biconcave deformities were from 12^th ^thoracic and below, in women also from the 11^th ^thoracic (Figure 3A and 3B).

**Table 3:** Distribution of numbers of deformities in women and men

	Men (N = 1206)		Women (N = 1681)	
Numbers of deformities	% (N)	95% CI (%)	% (N)	95% CI (%)
At least 1 deformity	9.7 (117)	8.0, 11.	7.8 (131)	46.5, 9.1
2 deformities	3.0 (36)	2.0, 4.0	2.5 (42)	1.8, 3.2
3 deformities or more	1.0 (13)	0.4, 1.6	1.4 (26)	0.8, 2.0

All	13.8 (166)	11.9, 15.8	11.8 (199)	10.4, 13.5

**Table 4:** Types of deformities at any vertebral level in 1681 women and 1206 men

	Men	Women
Types of deformities	N (%)	N (%)
**Wedge**	**141 (60.2)**	**162 (51)**
Mild wedge	0 (0)	0 (0)
Moderate wedge	92 (39)	86 (27)
Severe wedge	49 (21)	76 (24)
**Biconcave**	**78 (33.4)**	**116 (37)**
Mild biconcave	0 (0)	7 (2)
Moderate biconcave	49 (21)	62 (19.5)
Severe biconcave	29 (12.5)	47 (15)
**Compression**	**15 (6.4)**	**39 (12)**
Mild compression	0 (0)	0 (0)
Moderate compression	13 (5.5)	30 (9.5)
Severe compression	2 (1)	9 (3)

*Types of deformities differed significantly between the sexes (*p *= 0.025)

Finally, we examined the association between types of deformities and BMD at the total hip. In both sexes, BMD was significantly lower in persons with deformities compared to those without (*p *< 0.001). BMD also differed significantly according to type of deformity (ANOVA: *p *= 0.02 in women, *p *= 0.04 in men). In those with only one type of deformity observed, the age-adjusted mean total hip BMD was 0.855, 0.801 and 0.802 g/cm^2 ^in women, and 0.998, 0.950 and 0.935 in men, with wedge, biconcave and compression deformities, respectively.

## Discussion

The main finding in this study is that age was a significant predictor of vertebral deformities in both women and men with a prevalence increasing from approximately 3% in the age group below 60 years to approximately 20% in the age group 70+ in women, and from approximately 7.5% to 20% in men, respectively.

The rates of hip and forearm fractures in Norway are among the highest in the world. Because of this, one would expect the occurrence of vertebral fractures to be high as well. A surprising finding from this study is that this is not the case. For women, it can even be regarded as rather low compared to other studies [25,43], reported from Vietnam to be from 17.1% in the age group 50-59 to 39.2% in the age group 70+ (overall prevalence 23%) and in Spain from 7.2% in the age group 55-59 and 46.3% in the age group 75+ (overall 21.4%). In men, our results are more similar to those reported by others [24,26], prevalence being 4.7% in the age group 60-69, 10% in the 70-79 group, 14.6% in the 80+ group in Australia, and among Mexican men, 2% in age group 50-59 rising to 21.4% in the 80+ group, with an overall prevalence score of 9.7%. A multinational, European study from 1996 [45] found the overall prevalence to be 12% both in women and men, which is very much the same as in our study, but the Norwegian rates reported in that study were 19.2% in women, 15.7% in men, along with Sweden the highest rates in Europe. These Norwegian data were, however, extracted from a small sample (289 men, 298 women), mean age 65 years. In addition, another technology was used, making comparison difficult.

As reported by others, we also find the prevalence of vertebral deformities to be highest in the midthoracic region (5^th^-9^th ^thoracic) and thoracolumbar transition [42]. Wedge deformities were mostly found in the higher thoracic and the biconcave in the lower thoracic and lumbar region. It has been reported that fracture related disability may be greater among patient with lumbar fractures [42]. This could not be verified in the present study, but biconcave deformities were associated with lower BMD at the femoral sites in both sexes compared to the wedge deformities, suggesting a higher degree of severity. However, there is no consensus in the literature concerning type of vertebral fracture and severity [9]. The finding that prevalent radiographic vertebral fractures, of any type, are associated with low BMD measured at the femoral sites is reported by others [42]. As no X-rays were available in our study, we were unfortunately unable to assess whether the observed vertebral deformities are related to osteoporosis or other causes.

The Tromsø Study is a population-based, longitudinal study with a high participation rate. The present study is a cross-sectional survey within the framework of the Tromsø Study, where vertebral fracture assessments (VFA) were done for the first time. The intra-class correlation coefficient showed good reproducibility, indicating high methodological precision. Limitations of this study are that only prevalence data on vertebral deformities are presently available, also vertebral deformities were identified by DXA scanning only. Quality control of our data with x-rays on a sub-group was not possible within the scope of the survey. It is, however, reported that DXA scans are more precise in measuring moderate and severe than mild deformities [20]. Because of the methodological uncertainty concerning detection of mild deformities, the prevalence reported from our study may therefore be under-estimations. To address the issue of selection bias, we compared central characteristics between women and men who were randomly selected to either total body (TB) measurements (960 persons) or to the VFA (2894). In the VFA group, 58% were female compared to 62% in the TB group, with an OR of 1.21 (95% CI 1.04, 1.41), adjusted for age 1.22 (95% CI 1.05, 1.42). In the VFA group, both women and men were younger (65.4 versus 67.5 years in women, 65.3 versus 68.6 years in men), taller (162.2 versus 161.2 cm in women, 175.3 versus 174.4 cm in men), and men in the VFA group were also heavier (84.3 versus 82.3 kg) compared to men in the TB group. BMD levels at the total hip and femoral neck, health status, educational level and physical activity level did not differ between the groups. Despite the random selection, the VFA group was younger with a slightly higher proportion of women. However, when we compare the VFA group with the remaining phase 2 participants of the Tromsø VI survey, whom to our best knowledge should be a representative sample [37], the VFA sample of women and men was slightly older (3 years) and shorter (2 cm), but did not differ significantly in any other way. To summarize: we believe that the representativity of our sample is fair.

Throughout the study, we have deliberately used the term "vertebral deformity", though regarding these deformities as vertebral fractures [12,46]. Interestingly, the prevalence of vertebral fractures in the Tromsø population, which is considered a representative Norwegian population [38], does not follow the trend reported for non-vertebral fractures [35,36]. Difference in fracture mechanisms may possibly explain the discrepancy in prevalence, as non-vertebral fractures are connected to falls [47,48], whereas vertebral fractures are not [5]. One possible interpretation of the findings from this study is that the prevalence of vertebral fractures was low because our population was generally healthy and because of possible underestimations of mild deformities with the technology used. It has been reported that a large amount of vertebral fractures are asymptomatic [11]. Further studies should elaborate if physical function, pain and self-perceived health, as well as comorbidities, differ between persons with and without vertebral fractures.

## Conclusions

Although Norway reportedly has one of the highest incidences of forearm and hip fractures in world, data from the population-based Tromsø Study indicate that the prevalence of vertebral fractures, which increases by increasing age, is not higher than reported from other populations. The wedge fractures which most frequently occur in the thoracic region are the most common in both sexes. BMD was significantly lower in persons with vertebral fractures compared to those without.

## Competing interests

The authors declare that they have no competing interests with regard to this work.

## Authors' contributions

Contributions of the authors to the manuscript included *Study Concept and design: *NE, SW, AJS; *Aquistion of data: *NE; SW, NDN, LAA; *Analysis and interpretation of data: *SW, NE, LAA, AJS, JC, JAE; *Statistical analyses: *SW, NE, TN; *Critical revision of the manuscript: *SW, LAA, AJS, BM, JC, NDN, TN, JAE, NE. All authors read and approved the final manuscript.

## Pre-publication history

The pre-publication history for this paper can be accessed here:

http://www.biomedcentral.com/1471-2474/13/3/prepub
